# Diverse respiratory viruses detected among hospitalized patients with pneumonia in Sri Lanka and Vietnam

**DOI:** 10.1016/j.ijregi.2025.100757

**Published:** 2025-09-10

**Authors:** Phuong Thu Phan, Gaya Wijayaratne, Champica K. Bodinayake, Judith U. Oguzie, Thang Nguyen-Tien, Lyudmyla V. Marushchak, Jessica Rodriguez, Claudia M. Trujillo-Vargas, Ismaila Shittu, Nga Thanh Pham, Sen Thi Le, Ajith Nagahawatte, Phuong Thai Truong, Ruvini Kurukulasooriya, Armstrong Obale, Emily R. Robie, Emily S. Bailey, Bradly P. Nicholson, Christopher W. Woods, Gayani L. Tillekeratne, Gregory C. Gray

**Affiliations:** 1 Respiratory Center, Bach Mai Hospital, Hanoi, Vietnam; 2 Department of Microbiology, Faculty of Medicine, University of Ruhuna, Galle, Sri Lanka; 3 Department of Medicine, Faculty of Medicine, University of Ruhuna, Galle, Sri Lanka; 4 Duke Global Health Institute, Durham, USA; 5 Division of Infectious Diseases, Department of Internal Medicine, University of Texas Medical Branch, Galveston, USA; 6 Department of Microbiology, Bach Mai Hospital, Hanoi, Vietnam; 7 Duke-Ruhuna Collaborative Research Centre, Faculty of Medicine, University of Ruhuna, Galle, Sri Lanka; 8 Division of Infectious Diseases and International Health, Duke University School of Medicine, Durham, USA; 9 Campbell University, Buies Creek, USA; 10 Institute for Medical Research, Durham Veterans Affairs Medical Center, Durham, USA; 11 Department of Microbiology and Immunology, University of Texas Medical Branch, Galveston, USA; 12 Institute for Human Infections and Immunity, University of Texas Medical Branch, Galveston, USA

**Keywords:** CCoV-HuPn-2018, Sri Lanka, Vietnam, Pneumonia, Epidemiology, Emerging respiratory viruses

## Abstract

**Objectives:**

We conducted surveillance for novel respiratory viruses among patients hospitalized with pneumonia in Sri Lanka and Vietnam.

**Methods:**

We enrolled patients from January 2020 to July 2022 at one major hospital each in Sri Lanka and Vietnam. The participants’ nasopharyngeal swabs were studied with assays for previously recognized viruses as well as for assays for novel viruses.

**Results:**

A total of 401 hospitalized patients were enrolled, 204 from Sri Lanka and 197 from Vietnam. Laboratory analyses detected a number of previously recognized viruses, including influenza A/(H1N1)pdm09, human respiratory syncytial virus B, human respirovirus 3, rhinovirus A59, enteroviruses, and a human mastadenovirus C. Although an assortment of common human coronaviruses was characterized, 18 (43.9%) of 41 coronavirus-positive samples from Vietnam had evidence of the newly described canine-like coronavirus, CCoV-HuPn-2018 (previously only detected in Malaysia and Haiti).

**Conclusions:**

The detection of a novel, possibly emerging virus, CCoV-HuPn-2018, in multiple patients in Vietnam, demonstrates the value of such periodic novel virus surveillance, especially in regions at risk for viral emergence.

## Highlights

• We conducted surveillance for novel viruses in studying 401 patients hospitalized with pneumonia.
• Among these patients, we identified a variety of previously recognized respiratory viruses.
• Eighteen Vietnamese patients had evidence of a novel coronavirus (CCoV-HuPn-2018).
• Our findings underscore the importance of periodic surveillance for novel respiratory viruses.

## Introduction

During the past several decades, the world has experienced the emergence and spread of numerous novel respiratory viruses that have caused considerable morbidity and death [1]. Epidemiologists, ecologists, and modelers have worked to identify risk factors associated with emerging infectious diseases [2] and emerging zoonotic diseases [3]. The identified risk factors for specific viruses are complex and sometimes disparate; however, they often point to densely populated geographical regions as areas of high emerging infectious disease risk.

As previously published in considerable detail, we developed a strategy [1,4] to conduct surveillance for such novel respiratory viruses. The strategy calls for using pan-species conventional polymerase chain reaction (PCR) assays in conducting surveillance for novel viruses in six viral families. Often, we conduct this surveillance in a One Health way where humans frequently mix with large populations of animals [[5], [6], [7]]. Alternatively, we apply these viral discovery algorithms in studying patients hospitalized with pneumonia in geographical areas thought to be hot spots for viral emergence [8,9]. In this report, we summarize our novel respiratory virus surveillance efforts among patients with pneumonia at National Hospital Galle in Galle, Sri Lanka, and at Bach Mai Hospital in Hanoi, Vietnam.

## Methods

### Study design, subjects, and setting

National Hospital Galle (1800 beds) is the largest tertiary care center in Southern Sri Lanka. It is the main hospital for the Faculty of Medicine, University of Ruhuna. Established in 1982, the hospital offers free outpatient and inpatient services, including medical, pediatric, and surgical care, to an estimated 3.5 million people. National Hospital Galle covered the current study activities through a larger pneumonia etiology study called Respiratory Infection Severity and Etiology (RISE). All regulatory approvals were maintained under this umbrella.

Being one of the largest hospitals in Vietnam (1900 beds), Bach Mai Hospital in Hanoi is a national referral hospital that was established in 1911. It is one of only three highly specialized medical centers in Vietnam.

We conducted a cross-sectional study from January 2020 to August 2021 in Sri Lanka and from June 2021 to July 2022 in Vietnam. Potential participants were invited to enroll in the study via written informed consent. We included patients 1 year of age and above in Sri Lanka and patients of any age in Vietnam if they met all of the following criteria: they were admitted to a participating hospital, had evidence of acute infection (such as reported fever or chills, documented fever or hypothermia, leukocytosis or leukopenia, or new altered mental status), and had evidence of acute lower respiratory illness (including new cough or sputum production, chest pain, dyspnea, tachypnea, abnormal lung examination, or respiratory failure). Patients were excluded if they had been recently hospitalized, defined as within the past 28 days for immunocompetent individuals or within 90 days for immunosuppressed individuals. Additional exclusion criteria included prior enrollment in the study within the previous 28 days, the presence of a clear alternative diagnosis that explained the respiratory symptoms, such as asthma or chronic obstructive pulmonary disease exacerbation, metabolic acidosis, or heart disease, or structural abnormalities of the nasal anatomy that could increase the risk of bleeding or trauma during nasopharyngeal (NP) swab collection. In Sri Lanka, due to the COVID-19 prevention and control policies during the study period, patients were pre-screened for SARS-CoV-2 before enrollment. Patients with confirmed COVID-19 were admitted to separate isolation hospitals.

Enrolled patients completed a standardized questionnaire, and clinical staff collected an NP swab. Staff immediately transferred NP swabs into viral transport media and followed by specimen storage at –80°C. Routine pathogen assessments were conducted at the patients’ hospital or at Durham Veterans Affairs Medical Center; University of Texas Medical Branch (UTMB) supplemented these assays with pan-species assays for novel virus detection [1].

Sri Lankan investigators shipped the NP swabs to the Molecular Epidemiology Research Laboratory (MERL) of the Durham Veterans Affairs Medical Center in Durham, North Carolina, where they were studied with the NxTAG Respiratory Pathogen Panel (RPP) on the Luminex MAGPIX Integrated System platform (Luminex Corp., Austin, TX), which detects 19 viruses (adenovirus, coronavirus 229E, HKU1, NL63, OC43, human metapneumovirus, influenza A, influenza A-H1, influenza A–H1pdm09, influenza A-H3, influenza B, parainfluenza 1-4, respiratory syncytial virus A & B, rhinovirus/enterovirus, SARS-CoV-2), and two bacteria (*Chlamydia pneumoniae, Mycoplasma pneumoniae*), as well as the Centers for Disease Control and Prevention SARS-CoV-2 assay on an AB7500 Fast DX (Applied Biosystems).

In Vietnam, most of the NP swab samples were screened for influenza A & B and respiratory syncytial virus (RSV) A & B viruses with quantitative reverse transcription-PCR (qRT-PCR) assays at Bach Mai Hospital. Specimens were also screened at Bach Mai Hospital with a qRT-PCR SARS-CoV-2 assay.

Subsequently, NP swab specimens from both Sri Lanka and Vietnam were shipped to the UTMB at Galveston, Texas, USA, for novel pathogen discovery assay work.

### Supplemental laboratory analysis

#### Nucleic acid extraction

At UTMB, RNA and DNA were extracted from the NP swabs using QIAamp Viral RNA Mini Kit (Qiagen, Valencia, CA) for RNA extraction and the QIAamp DNA Mini Kit (Qiagen) for DNA extraction, following the manufacturer's instructions on the QIAcube Connect extraction instrument (Qiagen). For RNA and DNA extractions, 140 µl and 200 µl aliquots of NP swab specimens were used, respectively.

### PCR

#### Influenza A, B, and D qRT-PCR

After receiving the samples from both countries, at UTMB, all NP swab samples were screened for influenza A and B viruses using a World Health Organization (WHO) protocol [10], as well as for influenza D virus using an assay targeting the nucleoprotein gene [11]. Samples positive for influenza A were studied with conventional RT-PCR assays for virus subtypes using recommended protocols [12,13].

#### Pan-species PCR assays

To detect possible coronaviruses, a nested RT-PCR assay targeting the RNA-dependent RNA polymerase (RdRp) gene was performed [14]. The assay included an initial reverse transcription step using the SuperScript III Reverse Transcriptase (Invitrogen, USA), followed by a semi-nested PCR protocol. This assay has previously identified novel coronaviruses in humans [15] and cattle [16].

For pneumoviruses, conventional PCR targeting conserved motifs within the RdRp gene was used to screen the NP samples [17]. Detection of paramyxoviruses was performed using a pan-species, semi-nested RT-PCR assay targeting a conserved region of the RNA polymerase (L) gene, which broadly covers viruses across the family, subfamily, and genera of paramyxoviruses [18].

For enterovirus, a WHO enterovirus nested RT-PCR assay was used that targeted the VP1 gene of enteroviruses [19]. Briefly, RNA was first reverse transcribed using four primers, and the resulting cDNA served as the template for a nested PCR.

Finally, samples were tested for the presence of adenoviruses using a nested PCR assay targeting the adenovirus DNA polymerase gene, for both human and animal adenoviruses [20]. This assay previously detected a novel bovine adenovirus in a patient with pneumonia in Pakistan (unpublished personal communication GCG).

### Cell culture

We attempted to isolate viruses from the NP samples that had molecular evidence of CCoV-HuPn-2018 using two cell lines: A-72 (canine fibroblast cells; ATCC CRL-1542) and LLC-MK2 (rhesus monkey kidney cells; ATCC CCL-7). The LLC-MK2 cells were propagated in Eagle’s Minimum Essential Medium (EMEM; ThermoFisher Scientific, Gibco, cat. no. 11095-080), while A-72 cells were cultured in Leibovitz’s L-15 medium (ThermoFisher Scientific, Gibco, cat. no. 11415-064). All growth media were supplemented with 10% (v/v) fetal bovine serum (ThermoFisher Scientific, cat. no. 26140-079) and 1% (v/v) penicillin–streptomycin (ThermoFisher Scientific, cat. no. 15140-122). For each sample, a 20% (v/v) suspension of the NP swabs in serum-free maintenance medium was made and filtered using a 0.45 µM pore-size filter (Millipore Sigma™, cat no. SLHV033RS). The maintenance media consisted of EMEM and L-15 supplemented with 1X penicillin–streptomycin. For each type of maintenance medium, formulations were prepared with 2 µg/ml of tosyl phenylalanyl chloromethyl ketone-treated trypsin (TPCK-trypsin; Sigma-Aldrich, cat. no. 4352157-1KT) and inoculated on the monolayer of A-72 and LLC-MK2 cells. Inoculated cells were incubated at 37°C with 5% CO_2_. Cells were monitored for cytopathic effects every 24 hours over 8 days. At 3-4 days post-inoculation, the media were collected, and the cells were refed with fresh media. At 8 days post-inoculation, the infected cells and media were harvested, and RNA was extracted using the QIAamp Viral RNA Mini Kit (Qiagen, Valencia, CA).

### Next-generation sequencing and bioinformatics

Several NP swabs with molecular evidence of canine-like coronaviruses (CCoV) were selected for further characterization by next-generation sequencing (NGS). Sequencing libraries were constructed using the NEBNext Ultra II RNA Library Prep Kit for Illumina (New England Biolabs, Ipswich, MA) according to the manufacturer's specifications. Paired-end (75 bp) sequencing was performed on the NextSeq 550 Illumina platform (Illumina, Inc., San Diego, CA). Host backgrounds were depleted, and taxon classification was carried out using the CZID platform available at https://czid.org/.

### Phylogenetic analysis

A maximum likelihood phylogenetic tree was reconstructed to assess the evolutionary relationships of the detected sequences. Representative CCoV sequences, including those previously identified in human and animal hosts, were retrieved from the National Center for Biotechnology Information database. Multiple sequence alignment was conducted using MAFFT [21], and the phylogenetic tree was inferred using IQ-TREE [22] with the best-fit substitution model selected automatically. The resulting tree was visualized using FigTree v1.4.4 (http://tree.bio.ed.ac.uk/software/figtree/).

### Data analysis

Questionnaire and laboratory data were linked and examined for risk factors for specific viral causes of pneumonia. Counts and percentages were used for descriptive statistical analyses. Multivariate logistic regression modeling for evidence of CCoV-HuPn-2018 coronavirus by molecular assays at UTMB’s One Health laboratory was performed using R version 4.4.1. For the modeling, we first examined bivariate risk factor associations with Chi-square or Fisher’s exact tests, calculating odds ratios with 95% confidence intervals, including age, gender, occupation, and living location (urban, suburban, rural). Potential risk factors with a bivariate test statistic *P* <0.05 were included in a stepwise, backward elimination, unconditional logistic regression model. Risk factors with *P* <0.05 were retained in the final logistic regression models.

## Results

We studied 401 participants with pneumonia from the two sites, 204 patients from Sri Lanka and 197 patients from Vietnam (Table 1).

**Table 1:** Demographic characteristics of hospitalized patients with pneumonia.

Demographic characteristics	Sri Lanka N = 204		Vietnam N = 197		Total N = 401	
	n	%	n	%	n	%
**Sex**						
Male	116	56.9	84	42.6	200	49.9
Female	88	43.1	113	57.4	201	50.1
**Age group**						
≤17 years old	11	5.4	59	30.1	70	17.5
18-59 years old	99	48.5	73	37.2	172	43.0
≥60 years old	94	46.1	64	32.7	158	39.5
**Living location**						
Urban	47	23.3	61	34.1	108	28.3
Suburban	67	33.2	46	25.7	113	29.7
Rural	88	43.5	72	40.2	160	42.0

Note: Living location was missing for two participants from Sri Lanka and 18 from Vietnam; Age group was missing for one participant from Vietnam.

From Sri Lanka, male patients accounted for 56.9% of participants. Approximately half (48.5%) of the patients were aged 18-59 years, followed by those ≥60 years (46.1%). The remaining were children under 17 years of age (5.4%). Most of the patients (43.5%) were from rural areas. The NxTAG RPP results indicated that 18 out of 204 participants had evidence of respiratory viruses (Table 2). Rhinoviruses or enteroviruses were detected in 14 (6.8%) of the participants. One sample was positive for parainfluenza 1 and another one sample was positive for influenza A/H1pdm09. The remaining two samples were positive for coronavirus OC43.

**Table 2:** Molecular assays performed on nasopharyngeal swab specimens from participants hospitalized with pneumonia in Sri Lanka.

Durham Veterans Affairs Hospital NxTAG RPP	n = 204	UTMB One Health Laboratory Viral detection Molecular assays	n = 204
Influenza A	0	qRT-PCR Influenza A	Influenza A/(H1N1)pdm09, 1 (0.49%)
Influenza A-H1	0		
Influenza A-H3	0		
Influenza A–H1pdm09	1 (0.49%)		
Influenza B	0	qRT-PCR Influenza B	0
–		qRT-PCR Influenza D	0
SARS-CoV-2	0	Pan-*coronaviridae*	SARS-CoV-2, 1 (0.49%) Coronavirus OC43, 1 (0.49%)
Coronavirus 229E	0		
Coronavirus HKU1	0		
Coronavirus NL63	0		
Coronavirus OC43	2 (0. 98%)		
Rhinovirus/enterovirus –	14 (6.8%)	Pan-enterovirus	Rhinovirus A59, 1 (0.49%)
Adenovirus	0	Pan-*adenoviridae*	0
Parainfluenza 1	1 (0.49%)	Pan-*paramyxoviridae*	0
Parainfluenza 2	0		
Parainfluenza 3	0		
Parainfluenza 4	0		
Human metapneumovirus	0	Pan-*pneumoviridae*	0
RSV A	0		
RSV B	0		

The NxTAG RPP includes real-time PCR diagnostics for adenovirus, coronavirus 229E, HKU1, NL63, OC43, human metapneumovirus, influenza A virus, influenza A-H1 virus, influenza A-H3, influenza A–H1pdm09 virus, influenza B virus, parainfluenza viruses 1-4, respiratory syncytial virus A & B, rhinovirus/enterovirus, SARS-CoV-2, *Chlamydia pneumoniae*, and *Mycoplasma pneumoniae*.

RPP, respiratory pathogen panel; RSV, respiratory syncytial virus; qRT-PCR, quantitative reverse transcription-polymerase chain reaction; UTMB, University of Texas Medical Branch.

Among the 197 participants from Vietnam, 42.6% were male. Adults aged 18-59 years accounted for the highest percentage (37.2%) of total participants, followed by those ≥60 years (32.7%). In addition, a high percentage of the participants came from rural areas (40.2%). The molecular results at Bach Mai Hospital (Table 3) suggested that 4.1%, 6.5%, and 30.9% of 123 participants with pneumonia were infected with RSV A, RSV B, and SARS-CoV-2, respectively.

**Table 3:** Molecular assay results performed on nasopharyngeal swab specimens from participants hospitalized with pneumonia in Vietnam.

Bach Mai Hospital Assays	n = 123^a^	UTMB One Health Laboratory Viral detection Molecular assays	UTMB One Health Laboratory n = 197
qRT-PCR Influenza A	0	qRT-PCR Influenza A	0
qRT-PCR Influenza B	0	qRT-PCR Influenza B	0
–		qRT-PCR Influenza D	0
qRT-PCR RSV A	5 (4.1%)	Pan-*pneumoviridae*	RSV-B, 2 (1.0%)
qRT-PCR RSV B	8 (6.5%)		
qRT-PCR SARS-CoV-2	38 (30.9%)	Pan-*coronaviridae*	CCoV-HuPn-2018, 18 (9.1%) SARS-CoV-2, 22 (11.1%) Coronavirus OC43, 1 (0.5%)
–		Pan-enterovirus	5 (2.5%)
–		Pan-*paramyxoviridae*	HPIV-3, 5 (2.50%)
–		Pan-*adenoviridae*	Human mastadenovirus C, 1 (0.50%)

HPIV-3, human parainfluenza virus 3 or human respirovirus 3; qRT-PCR, quantitative reverse transcription-polymerase chain reaction; RSV, respiratory syncytial virus; UTMB, University of Texas Medical Branch;

a Due to lack of reagents, Bach Mai Hospital did not run influenza A or RSV molecular assays on all participants.

### Laboratory results at UTMB

The UTMB assays indicated that one specimen was positive for influenza A virus in Sri Lanka with a cycle threshold (Ct) value of 34.37 according to the qRT-PCR assay targeting the M gene (Table 2), and this was confirmed as influenza A subtype H1pdm09 by the WHO qRT-PCR protocol [12], with a Ct value of 33.51. We did not detect influenza B or D viruses in any of the NP swab specimens from either country.

**Figure 1:**
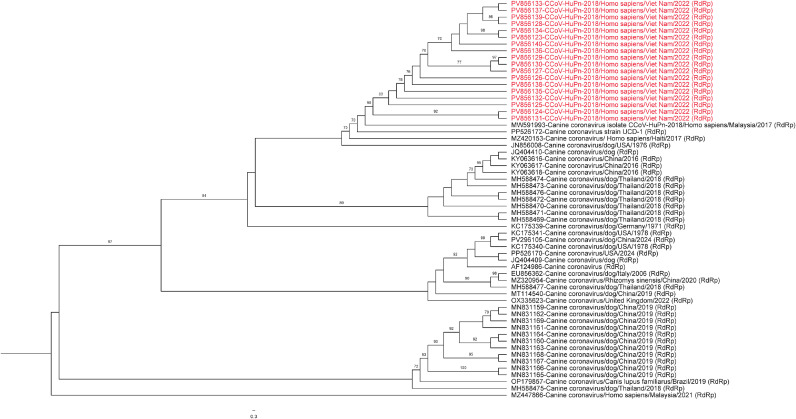
This phylogenetic tree illustrates the very close relationship of the Sanger sequences generated by pan-coronavirus assays derived from the nasopharyngeal swabs of 18 patients with pneumonia at Bach Mai Hospital in Vietnam. When these sequences were compared with the first detected human isolate of CCoV-HuPn-2018 Malaysia [9], identity scores ranged from 99.6-99.8%. They were also very closely related to the very similar virus detected in a human with a respiratory illness returning from Haiti in 2017.

At UTMB, molecular evidence of coronavirus infection was detected in two (0.98%) specimens from Sri Lanka (Table 2) and 41 (20.8%) specimens from Vietnam (Table 3). Via Sanger sequencing, we found that 22/41 (53.6%) from Vietnam and one sample from Sri Lanka had molecular evidence of SARS-COV-2. Remarkably, 18/41 (43.9%) in Vietnam were found by Sanger sequencing to be very similar phylogenetically **(**Figure 1) to the human canine-like coronavirus, CCoV-HuPn-2018, with sequence identity scores ranging from 99.6-99.8% compared with the first detected human CCoV-HuPn-2018 isolate from Malaysia [15] (GenBank accession number MW591993). In addition, two NP swab samples (one from Vietnam and one from Sri Lanka) had molecular evidence of human coronavirus OC43.

At UTMB, one NP swab sample from Sri Lanka had molecular evidence of enterovirus, and this specimen was further characterized as rhinovirus A59. In Vietnam, five samples had evidence for enterovirus (Table 3). However, the bands were too faint for characterization by Sanger sequencing. A supplemental enterovirus qRT-PCR assay [23] confirmed one positive sample out of the five identified, with a Ct value of 29.93. One of those five samples also had molecular evidence of SARS-COV-2.

All samples from Sri Lanka were negative for the pan-*pneumoviridae* assay. However, two samples from Vietnam showed positive results. Sanger sequencing suggested that these two specimens were likely RSV B. The pan-*paramyxoviridae* assay was also negative for all NP swab samples from Sri Lanka, but seven Vietnamese samples yielded bands of the correct molecular weight. Five of these samples were identified as human respirovirus 3 with Ct values ranging from 16.28-25.31. The presence of human respirovirus 3 was further confirmed in all five specimens using a supplemental qRT-PCR assay for HPIV-3 [24]. Interestingly, one sample was also found to have evidence of pan-*pneumoviridae*, suggesting a dual infection with both HPIV-3 and RSV B.

Only one sample from Vietnam was positive by the pan-*adenoviridae* assay, and this specimen’s sequence data suggested a human mastadenovirus C was present.

Regarding the Sri Lankan specimens, both the NxTAG RPP and UTMB assays identified that one NP swab had evidence of influenza A, specifically influenza A/(H1N1)pdm09 virus. In addition, both the NxTAG RPP and UTMB assays agreed that one NP swab had evidence of a rhinovirus and another NP sample had evidence for coronavirus OC43. However, there were a number of discordant results between the NxTAG RPP and UTMB assays (Table 2). Similarly, there were a number of discordant results between Bach Mai Hospital and UTMB assay testing (Table 3). We were not successful in isolating canine coronavirus from any of the 18 NP swabs with molecular evidence of CCoV-HuPn-2018 using A72 and LLC-MK2 cell lines.

### Next-generation sequencing

We performed metagenomic NGS on six PCR-positive samples for coronavirus in Vietnam to further characterize viral genomes initially detected by Sanger sequencing. CCoV-HuPn-2018 was identified in multiple samples **(**Figure 1), and they were closely related to CCoV-HuPn-2018 detections in Malaysia [15] and Haiti [25]. However, the coverage was insufficient to support complete genome assembly. We were successful in assembling a near-complete genome of rhinovirus A61 (99.9% genome coverage) from one sample from Vietnam. In addition, partial genome assembly of enterovirus A71 was achieved from another sample from Vietnam, with approximately 38% genome coverage.

### Risk factor analyses

We examined potential risk factors for the evidence of CCoV-HuPn-2018 in Vietnam only. However, we found no statistically significant risk factors for molecular evidence of CCoV-HuPn-2018 modeling for Vietnam.

## Discussion

We found evidence of diverse viral infections in NP swabs of patients with pneumonia in Sri Lanka and Vietnam using molecular assays for previously recognized respiratory viruses, as well as pan-*coronaviridae*, pan-enterovirus, pan-*pneumoviridae*, pan-*paramyxoviridae*, and pan-*adenoviridae* with Sanger sequencing. We further studied some novel detections with cell culture and NGS.

Although diversity was high, the prevalence of specific viruses, except for SARS-CoV-2 and CCoV-HuPn-2018, was low, compared with another similar study we conducted in Sarawak, Malaysia (under journal review), where among 441 pneumonia patients without evidence of SARS-CoV-2, 78.2% had at least one virus detected and 24.9% had multiple viruses detected. It seems likely that viral activity other than SARS-CoV-2 was suppressed with pandemic social distancing measures and other precautions observed during the SARS-CoV-2 pandemic. However, we cannot exclude other hypotheses, such as the patients in these cohorts were more likely to have bacterial infections or infections due to viruses not captured by our assays. In addition, it should be noted that during this time period, patients in Sri Lanka with SARS-CoV-2 were often sent to separate isolation hospitals. Hence, the low viral counts of Sri Lankan specimens, including SARS-CoV-2, were likely related to the pandemic’s regulations and behavioral changes. Although before the pandemic, studies in Sri Lanka revealed a higher percentage of influenza A and B of 5-9% [26,27]. Nevertheless, the prevalence of other respiratory viral infections was still low using the Luminex Integrated System NxTAG RPP with 19 respiratory viruses [26]. In Hanoi, Vietnam, a previous study showed a low prevalence of coronavirus (1.1%) but high prevalence of influenza A (64.7%) and B (29.3%) among patients with severe acute respiratory illness [28]. However, this older study enrolled participants at different hospitals. Our findings support the value of performing surveillance for novel viral infections among patients hospitalized with pneumonia in areas at increased risk of novel pathogen emergence using low-cost pan-species assays and additional laboratory methods, including cell culture and sequencing analysis. Finding evidence from this surveillance strategy can help us detect viral pathogens missed by other molecular methods [9] or even characterize the novel respiratory viruses that may result in a future pandemic. In our study, the detection of CCoV-HuPn-2018 in multiple patients in Vietnam demonstrates the value of such surveillance. This finding is remarkable as CCoV-HuPn-2018 has only previously been detected in Malaysia [15] and Haiti [25]. As described before, there is now considerable evidence that this novel recombinant alphacoronavirus is the eighth unique coronavirus recognized to cause disease in humans [15]. Further epidemiological studies are required to better estimate the importance of this pathogen. Due to the very limited exposure data from these patients, we were not able to identify risk factors for its detection.

Our study had a number of limitations. We also found laboratory assay discordance between the two sets of laboratories studying both the Sri Lankan and Vietnamese specimens. This might be explained by the different assays, different laboratory procedures, freeze-thaw specimen degradation, etc. It was interesting to note that NxTAG RPP found more positive samples than our UTMB pan-species assays, especially for enteroviruses/rhinoviruses. The scientific literature suggests that NxTAG RPP remains generally less sensitive and specific than singleplex RT-PCR [29]. In addition, assays for influenza A & B, RSV A & B, and SARS-CoV-2 were not performed on 37% of 197 specimens in Vietnam (Table 3), so that we could not compare the effectiveness between those assays with the pan-species assays at UTMB. We also did not perform comprehensive analyses for bacterial causes of infection; thus, these viral etiology data do not represent the entire spectrum of pathogens that may be associated with the patients’ illness.

## Conclusion

The detection of CCoV-HuPn-2018 in multiple patients in Vietnam demonstrates the value of such surveillance. These were the first detection of CCoV-HuPn-2018 in Vietnam. Having detected this virus in three different countries (Malaysia, Haiti, and Vietnam), it now seems likely that this novel virus or similar viruses are much more widely geographically dispersed. These findings highlight the critical need for continued surveillance for new viral causes of pneumonia, especially in regions at risk for viral emergence.

## Declaration of competing interest

The authors have no competing interests to declare.

## Appendix Supplementary materials

Image, application 1
